# Di-μ-acetato-bis­[(acetato-κ^2^ *O*,*O*′)bis­(iso­nicotinamide-κ*N*)copper(II)]

**DOI:** 10.1107/S1600536810004393

**Published:** 2010-02-10

**Authors:** Mireille Perec, Ricardo Baggio

**Affiliations:** a Departamento de Química Inorgánica, Analítica y Química Física, INQUIMAE, Facultad de Ciencias Exactas y Naturales, Universidad de Buenos Aires, Ciudad Universitaria, Pabellón II, 1428 Buenos Aires, Argentina; b Departamento de Física, Centro Atómico Constituyentes, Comisión Nacional de Energía Atómica, Buenos Aires, Argentina

## Abstract

The title centrosymmetric bimetallic complex, [Cu_2_(C_2_H_3_O_2_)_4_(C_6_H_6_N_2_O)_4_], is composed of two copper(II) cations, four acetate anions and four isonicotinamide (INA) ligands. The asymmetric unit contains one copper cation to which two acetate units bind asymmetrically; one of the Cu—O distances is rather long [2.740 (2) Å], almost at the limit of coordination. These Cu—O bonds define an equatorial plane to which the Cu—N bonds to the INA ligands are almost perpendicular, the Cu—N vectors subtending angles of 2.4 (1) and 2.3 (1)° to the normal to the plane. The metal coordination geometry can be described as a slightly distorted trigonal bipyramid if the extremely weak Cu—O bond is disregarded, or as a highly distorted square bipyramid if it is not. The double acetate bridge between the copper ions is not coplanar with the CuO_4_ equatorial planes, the dihedral angle between the (O—C—O)_2_ and O—Cu—O groups being 34.3 (1)°, resulting in a sofa-like conformation for the 8-member bridging loop. In the crystal, N—H⋯O hydrogen bonds occur, some of which generate a head-to tail-linkage between INA units, giving raise to chains along [101]; the remaining ones make inter-chain contacts, defining a three-dimensional network. There are in addition a number of C—H⋯O bonds involving aromatic H atoms. Probably due to steric hindrance, the aromatic rings are not involved in significant π⋯π inter­actions.

## Related literature

For the importance of Cu(II) carboxyl­ate complexes in biology, see: Lippard & Berg (1994 ▶). For coordination properties of anionic carboxyl­ates, see: Deacon & Phillips (1980 ▶). For related compounds obtained from the same (or similar) reaction, see: Aakeröy *et al.* (2003 ▶). For a chloro­acetate analogue of the title compound, see: Moncol *et al.* (2007 ▶).
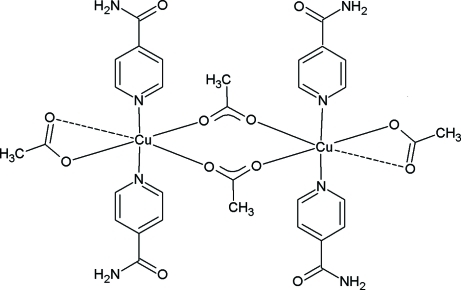


## Experimental

### 

#### Crystal data

[Cu_2_(C_2_H_3_O_2_)_4_(C_6_H_6_N_2_O)_4_]
*M* *_r_* = 851.77
Monoclinic, 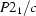

*a* = 10.910 (2) Å
*b* = 11.462 (2) Å
*c* = 15.644 (3) Å
β = 104.05 (3)°
*V* = 1897.6 (7) Å^3^
*Z* = 2
Mo *K*α radiation
μ = 1.19 mm^−1^
*T* = 294 K
0.28 × 0.18 × 0.14 mm

#### Data collection

Rigaku AFC6 Difractometer diffractometer
Absorption correction: ψ scan (North *et al.*, 1968 ▶) *T*_min_ = 0.76, *T*_max_ = 0.85
12257 measured reflections
3736 independent reflections
2384 reflections with *I* > 2σ(*I*)
*R*_int_ = 0.064
3 standard reflections every 150 reflections  intensity decay: <2%

#### Refinement

*R*[*F* ^2^ > 2σ(*F* ^2^)] = 0.039
*wR*(*F* ^2^) = 0.099
*S* = 1.08
3736 reflections
244 parameters
H-atom parameters constrained
Δρ_max_ = 0.36 e Å^−3^
Δρ_min_ = −0.43 e Å^−3^

### 

Data collection: *MSC/AFC Diffractometer Control Software* (Molecular Structure Corporation, 1988 ▶); cell refinement: *MSC/AFC Diffractometer Control Software*; data reduction: *MSC/AFC Diffractometer Control Software*; program(s) used to solve structure: *SHELXS97* (Sheldrick, 2008 ▶); program(s) used to refine structure: *SHELXL97* (Sheldrick, 2008 ▶); molecular graphics: *SHELXTL-NT* (Sheldrick, 2008 ▶); software used to prepare material for publication: *SHELXTL-NT* and *PLATON* (Spek, 2009 ▶).

## Supplementary Material

Crystal structure: contains datablocks I, New_Global_Publ_Block. DOI: 10.1107/S1600536810004393/hb5326sup1.cif

Structure factors: contains datablocks I. DOI: 10.1107/S1600536810004393/hb5326Isup2.hkl

Additional supplementary materials:  crystallographic information; 3D view; checkCIF report

## Figures and Tables

**Table 1:** Selected bond lengths (Å)

Cu1—O13	1.952 (2)
Cu1—O14	2.020 (2)
Cu1—N12	2.027 (2)
Cu1—N11	2.047 (2)
Cu1—O23^i^	2.271 (2)
Cu1—O24	2.740 (2)

Symmetry code: (i) 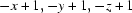.

**Table 2:** Hydrogen-bond geometry (Å, °)

*D*—H⋯*A*	*D*—H	H⋯*A*	*D*⋯*A*	*D*—H⋯*A*
N21—H21*A*⋯O12^ii^	0.86	2.03	2.884 (3)	174
N21—H21*B*⋯O14^iii^	0.86	2.15	2.946 (3)	154
N22—H22*A*⋯O11^iv^	0.86	2.10	2.955 (3)	174
N22—H22*B*⋯O24^v^	0.86	2.19	3.044 (4)	172
C22—H22⋯O24^v^	0.93	2.26	3.154 (4)	160
C11—H11⋯O23^i^	0.93	2.56	3.047 (4)	113
C12—H12⋯O23^i^	0.93	2.53	3.054 (4)	116
C51—H51⋯O13	0.93	2.36	2.910 (4)	117
C52—H52⋯O13	0.93	2.57	3.017 (4)	110

Symmetry codes: (i) 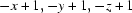; (ii) 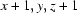; (iii) 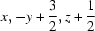; (iv) 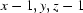; (v) 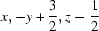.

**Fig. 1.**
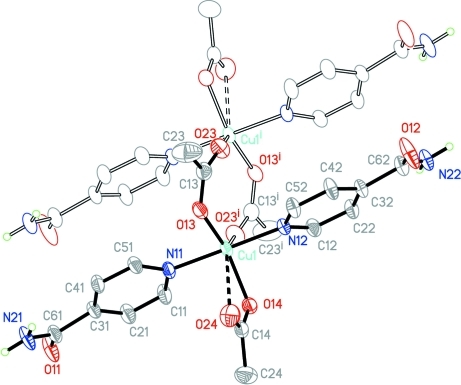
Molecular view of a dimer, with displacement ellipsoids at a 40% level. Atoms in the asymmetric unit drawn in full ellipsoids and full bonds; symmetry related ones (through the i: 1 - x, 1 - y, 1 - z operation), in empty ellipsoids and simple bonds. In double broken lines, the extremely weak Cu—O interaction. H atoms bound to carbon not shown, for clarity.

**Fig. 2.**
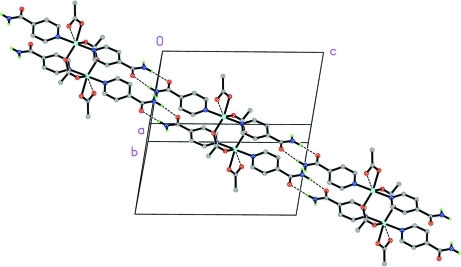
Schematic view of a chain running along [101]. Intrachain H-bonds drawn in broken lines. C—H atoms omitted for clarity.

## supplementary crystallographic information

### Comment

Lewis based coordinated Cu(II) carboxylate complexes are an important class of coordination compounds due to their relevance as structural and functional models for biologically important metalloenzymes (Lippard & Berg, 1994). Anionic carboxylates are highly flexible and versatile O-donor ligands since a range of substituents may be introduced on the alkyl chain to modulate its reactivity and coordination propensity and result in a variety of coordination modes such as monodentate, bidentate bridging, chelating, monoatomic bridging and chelating bridging (Deacon & Phillips, 1980). The Lewis base isonicotinamide acts as an effective tool for assembling coordination building units of Cu(II) into infinite 1-D chains. It has been reported that the reaction of Cu(II) acetate with isonicotinamide in acetonitrile (molar ratio 1:10) and drops of glacial acetic acid afforded the tetrakis (*µ*-acetato-*O*,*O*')-bis(isonicotinamide-*N*) dicopper (II) acetonitrile, whereas the same rection in methanol affords bis{bis(*µ*_2_-acetato-*O*)-aceticacid-*O*-bis(isonicotinamide-*N*)copper}bis(methanol) (Aakeröy *et al.*, 2003). The crystal structure of the former contains the classical "paddle-wheel" core and peripheral isonicotinamide ligands with the amides oriented linearly and pointing in opposite directions. In the latter, two monodentate acetates and two isonicotinamides are in a plane in *trans*- geometry with a third acetate completing a square-pyramidal arrangement, two acetates coordinate to neighbouring coppers in a *µ*_2 _coordination, creating the dinuclear species. We now report a third structure obtained from the reaction of isonicotinamide and Cu(II) acetate (1:1) in methanol, (C_16_H_18_CuN_4_O_6_)_2_(I). The structure here consists of a dinuclear unit with two bridging *µ*_2_ acetate ligands, two peripheral acetate ligands, and four axial isonicotinamide ligands. The common feature in the three structures with different Cu(II) coordination geometries is the role played by the isonicotinamide units as rigid structures to guide the direction of propagation of the hydrogen-bonded links in the 1-D constructions.

The dimeric title compound (I) (Fig. 1) is built up around a center of symmetry; the independent unit is composed of one cation to which two acetate units bind, both of them in rather asymmetric way: the one with trailing number 3, bridging both copper cations in a double bridge (Cu1—O13: 1.952 (2) Å; Cu1—O23^i^: 2.271 (2) Å, (i): 1 - *x*,1 - *y*,1 - *z*); the remaining one (trailing number 4) binding each cation in a chelating manner, with a Cu—O bond in the normal range (Cu1—O14: 2.020 (2) Å) and a second, extremely long contact almost in the limit of coordination (Cu1—O24: 2.740 (2) Å). These bonds define an equatorial plane to which the Cu—N bonds provided by the INA groups (Cu1—N11: 2.047 (2); Cu1—N12: 2.027 (2) Å) are almost perpendicular, the preceeding Cu—N vectors subtending angles of 2.4 (1) and 2.3 (1)° to the plane normal. The coordination geometry thus described could be defined as as lightly distorted trigonal bipyramid, if the weak Cu1—O24 bond is disregarded, or as a highly distorted square bipyramid, if not. The double acetate bridge is non-coplanar to the cation equatorial planes, the corresponding O—C—O and O—Cu—O planes forming a dihedral angle of 34.3 (1)° and resulting in a sofa-like conformation for the 8-member bridging loop.

The packing organization is governed by N—H···O interactions (Table 1, first 4 entries). Those involving H21*a* and H22*a* generate the characteristic head to tail linkage between INA units, giving raise to chains along [101] (Fig. 2). This particular disposition leaves H21*b* and H22*b* pointing outwards the chains, in a favourable disposition to make interchain contacts to define a strong three-dimensional network. There are in addition a number of C—H···O bonds involving aromatic H atoms. One of them (fifth entry in Table 1), the only one involving the bridging acetate, is rather strong for a non conventional H-bond and provides to interchain cohesion, while the remaining four, involving the chelating acetate O atoms, are intradimeric. Probably due to steric hindrance, the aromatic rings are not involved in significant π···πinteractions.

A chloroacetate isolog of the title compound has been recently described in the literature (Moncol *et al.*, 2007), and in spite of presenting an anisotropic cell expansion/contraction as compared to (I) (Unit cell differences: -1% in a, +5% in b, +1% in c) the general trend both in the dimer metrics as well as in packing interactions is extremely similar.

### Experimental

To a solution of Cu(CH_3_CO_2_)_2_.H_2_O (0.20 g, 0.01 mol) in methanol (40 cm^3^) at troom temperature was added solid isonicotinamide (INA) (0.14 g, 0.01 mol) in small portions under constant stirring. It was then filtered and the solution allowed to stand for two days, after which small blue blocks of (I) were filtered and dried under vacuum. Yield: 0.28 g (80%). Found: C, 45.10; H, 4.23; N, 13.20; Cu, 15.02%. Calc. for C_32_H_36_Cu_2_N_8_O_12_: C, 45.08; H, 4.26; N, 13.16; Cu, 14.92%.

### Refinement

All H atoms were located at idealized positions (C—H: 0.93 Å, N—H: 0.85 Å) after being confirmed by inspection in a difference map. They were allowed to ride, with U_iso_(H) = 1.2*U*_eq_(host)

### Crystal data

**Table:**

[Cu_2_(C_2_H_3_O_2_)_4_(C_6_H_6_N_2_O)_4_]	*F*(000) = 876
*M**_r_* = 851.77	*D*_x_ = 1.491 Mg m^−^^3^
Monoclinic, *P*2_1_/*c*	Mo *K*α radiation, λ = 0.71073 Å
Hall symbol: -P 2ybc	Cell parameters from 25 reflections
*a* = 10.910 (2) Å	θ = 7.5–15.0°
*b* = 11.462 (2) Å	µ = 1.19 mm^−^^1^
*c* = 15.644 (3) Å	*T* = 294 K
β = 104.05 (3)°	Block, blue
*V* = 1897.6 (7) Å^3^	0.28 × 0.18 × 0.14 mm
*Z* = 2	

### Data collection

**Table:**

Rigaku AFC6 Difractometer diffractometer	2384 reflections with *I* > 2σ(*I*)
Radiation source: fine-focus sealed tube	*R*_int_ = 0.064
graphite	θ_max_ = 26.0°, θ_min_ = 1.9°
ω/2θ scans	*h* = −3→13
Absorption correction: ψ scan (North *et al.*, 1968)	*k* = −13→14
*T*_min_ = 0.76, *T*_max_ = 0.85	*l* = −19→19
12257 measured reflections	3 standard reflections every 150 reflections
3736 independent reflections	intensity decay: <2%

### Refinement

**Table:**

Refinement on *F*^2^	Primary atom site location: structure-invariant direct methods
Least-squares matrix: full	Secondary atom site location: difference Fourier map
*R*[*F*^2^ > 2σ(*F*^2^)] = 0.039	Hydrogen site location: inferred from neighbouring sites
*wR*(*F*^2^) = 0.099	H-atom parameters constrained
*S* = 1.08	*w* = 1/[σ^2^(*F*_o_^2^) + (0.0438*P*)^2^] where *P* = (*F*_o_^2^ + 2*F*_c_^2^)/3
3736 reflections	(Δ/σ)_max_ = 0.001
244 parameters	Δρ_max_ = 0.36 e Å^−^^3^
0 restraints	Δρ_min_ = −0.43 e Å^−^^3^

### Special details

**Table:**

Geometry. All esds (except the esd in the dihedral angle between two l.s. planes) are estimated using the full covariance matrix. The cell esds are taken into account individually in the estimation of esds in distances, angles and torsion angles; correlations between esds in cell parameters are only used when they are defined by crystal symmetry. An approximate (isotropic) treatment of cell esds is used for estimating esds involving l.s. planes.

### Fractional atomic coordinates and isotropic or equivalent isotropic displacement parameters (Å^2^)

**Table:**

	*x*	*y*	*z*	*U*_iso_*/*U*_eq_	
Cu1	0.52008 (4)	0.66370 (3)	0.55107 (2)	0.03432 (14)	
N11	0.6436 (2)	0.6393 (2)	0.67105 (15)	0.0354 (6)	
N21	0.8631 (2)	0.6620 (3)	0.99514 (15)	0.0515 (8)	
H21A	0.9136	0.6681	1.0465	0.062*	
H21B	0.7827	0.6654	0.9894	0.062*	
O11	1.0221 (2)	0.6417 (3)	0.92761 (13)	0.0622 (8)	
C11	0.7678 (3)	0.6569 (3)	0.6801 (2)	0.0453 (8)	
H11	0.7968	0.6700	0.6297	0.054*	
C21	0.8542 (3)	0.6563 (3)	0.76043 (19)	0.0458 (8)	
H21	0.9396	0.6671	0.7635	0.055*	
C31	0.8135 (3)	0.6396 (3)	0.83658 (19)	0.0355 (7)	
C41	0.6858 (3)	0.6182 (3)	0.82759 (18)	0.0384 (8)	
H41	0.6548	0.6037	0.8770	0.046*	
C51	0.6054 (3)	0.6187 (3)	0.74488 (18)	0.0368 (7)	
H51	0.5201	0.6040	0.7399	0.044*	
C61	0.9091 (3)	0.6474 (3)	0.92481 (19)	0.0401 (8)	
N12	0.4021 (2)	0.6837 (2)	0.43010 (14)	0.0338 (6)	
N22	0.2051 (3)	0.6561 (2)	0.10087 (15)	0.0438 (7)	
H22A	0.1567	0.6508	0.0487	0.053*	
H22B	0.2852	0.6462	0.1091	0.053*	
O12	0.0434 (2)	0.6944 (3)	0.16185 (14)	0.0768 (10)	
C12	0.4499 (3)	0.6853 (3)	0.35855 (19)	0.0400 (8)	
H12	0.5373	0.6871	0.3671	0.048*	
C22	0.3768 (3)	0.6843 (3)	0.27344 (19)	0.0401 (8)	
H22	0.4146	0.6840	0.2261	0.048*	
C32	0.2462 (3)	0.6838 (3)	0.25892 (18)	0.0365 (8)	
C42	0.1959 (3)	0.6871 (3)	0.33279 (18)	0.0441 (9)	
H42	0.1089	0.6898	0.3260	0.053*	
C52	0.2757 (3)	0.6864 (3)	0.41562 (19)	0.0411 (8)	
H52	0.2403	0.6878	0.4641	0.049*	
C62	0.1560 (3)	0.6793 (3)	0.16855 (19)	0.0415 (8)	
O13	0.3889 (2)	0.5867 (2)	0.59603 (12)	0.0434 (6)	
O23	0.3372 (2)	0.4336 (2)	0.50649 (14)	0.0500 (6)	
C13	0.3202 (3)	0.5003 (3)	0.5648 (2)	0.0408 (8)	
C23	0.2067 (4)	0.4812 (4)	0.6042 (3)	0.0855 (14)	
H23A	0.1303	0.4859	0.5583	0.128*	
H23B	0.2056	0.5397	0.6478	0.128*	
H23C	0.2128	0.4054	0.6310	0.128*	
O14	0.61484 (19)	0.81068 (17)	0.53598 (12)	0.0355 (5)	
O24	0.4839 (2)	0.8831 (2)	0.61005 (15)	0.0558 (7)	
C14	0.5792 (3)	0.8918 (3)	0.58074 (19)	0.0372 (7)	
C24	0.6596 (4)	0.9995 (3)	0.5983 (2)	0.0635 (11)	
H24A	0.6678	1.0316	0.5433	0.095*	
H24B	0.7418	0.9798	0.6338	0.095*	
H24C	0.6209	1.0559	0.6287	0.095*	

### Atomic displacement parameters (Å^2^)

**Table:**

	*U*^11^	*U*^22^	*U*^33^	*U*^12^	*U*^13^	*U*^23^
Cu1	0.0350 (2)	0.0431 (2)	0.01981 (18)	−0.0048 (2)	−0.00328 (14)	0.00104 (18)
N11	0.0377 (15)	0.0443 (16)	0.0207 (12)	−0.0029 (12)	0.0005 (11)	0.0014 (11)
N21	0.0301 (14)	0.099 (2)	0.0211 (12)	−0.0010 (17)	−0.0028 (11)	−0.0061 (15)
O11	0.0350 (13)	0.123 (2)	0.0245 (11)	−0.0002 (15)	−0.0009 (10)	−0.0015 (14)
C11	0.0378 (18)	0.071 (2)	0.0250 (15)	−0.0064 (19)	0.0036 (14)	0.0053 (17)
C21	0.0341 (18)	0.071 (2)	0.0298 (16)	−0.0078 (19)	0.0032 (14)	0.0023 (17)
C31	0.0313 (16)	0.047 (2)	0.0242 (14)	−0.0024 (15)	−0.0003 (12)	−0.0017 (14)
C41	0.0389 (18)	0.052 (2)	0.0221 (14)	−0.0018 (16)	0.0029 (13)	0.0027 (14)
C51	0.0305 (16)	0.0485 (19)	0.0280 (15)	−0.0025 (15)	0.0001 (13)	−0.0011 (14)
C61	0.0353 (18)	0.053 (2)	0.0263 (15)	−0.0020 (17)	−0.0043 (14)	0.0014 (15)
N12	0.0358 (14)	0.0390 (16)	0.0226 (12)	−0.0013 (12)	−0.0006 (11)	−0.0003 (10)
N22	0.0381 (15)	0.0658 (18)	0.0227 (12)	0.0051 (15)	−0.0016 (11)	−0.0014 (13)
O12	0.0387 (14)	0.156 (3)	0.0292 (12)	0.0195 (17)	−0.0046 (11)	−0.0138 (15)
C12	0.0295 (16)	0.055 (2)	0.0314 (16)	−0.0034 (15)	0.0002 (13)	0.0027 (15)
C22	0.0387 (18)	0.057 (2)	0.0235 (14)	0.0013 (17)	0.0050 (13)	0.0008 (14)
C32	0.0352 (17)	0.048 (2)	0.0217 (14)	0.0035 (15)	−0.0016 (13)	−0.0017 (13)
C42	0.0326 (17)	0.068 (3)	0.0266 (15)	0.0103 (17)	−0.0026 (13)	0.0011 (15)
C52	0.0431 (19)	0.056 (2)	0.0232 (15)	0.0085 (16)	0.0058 (14)	−0.0014 (14)
C62	0.0376 (19)	0.056 (2)	0.0260 (15)	0.0064 (17)	−0.0008 (14)	−0.0003 (15)
O13	0.0447 (13)	0.0550 (15)	0.0267 (11)	−0.0126 (12)	0.0014 (10)	−0.0026 (10)
O23	0.0490 (14)	0.0535 (14)	0.0439 (13)	0.0058 (13)	0.0040 (11)	−0.0070 (12)
C13	0.0376 (17)	0.046 (2)	0.0364 (18)	0.0006 (17)	0.0046 (14)	0.0057 (17)
C23	0.075 (3)	0.077 (3)	0.116 (4)	−0.026 (3)	0.045 (3)	−0.020 (3)
O14	0.0361 (12)	0.0407 (13)	0.0288 (10)	−0.0035 (10)	0.0063 (9)	−0.0003 (9)
O24	0.0549 (16)	0.0677 (17)	0.0524 (14)	0.0021 (13)	0.0277 (13)	0.0018 (13)
C14	0.0390 (19)	0.0441 (19)	0.0263 (15)	−0.0015 (16)	0.0037 (14)	0.0035 (15)
C24	0.081 (3)	0.049 (2)	0.061 (2)	−0.013 (2)	0.018 (2)	−0.015 (2)

### Geometric parameters (Å, °)

**Table:**

Cu1—O13	1.952 (2)	N22—H22B	0.8600
Cu1—O14	2.020 (2)	O12—C62	1.220 (4)
Cu1—N12	2.027 (2)	C12—C22	1.376 (4)
Cu1—N11	2.047 (2)	C12—H12	0.9300
Cu1—O23^i^	2.271 (2)	C22—C32	1.387 (4)
Cu1—O24	2.740 (2)	C22—H22	0.9300
N11—C51	1.341 (4)	C32—C42	1.395 (4)
N11—C11	1.343 (4)	C32—C62	1.515 (4)
N21—C61	1.326 (4)	C42—C52	1.374 (4)
N21—H21A	0.8600	C42—H42	0.9300
N21—H21B	0.8600	C52—H52	0.9300
O11—C61	1.224 (4)	O13—C13	1.265 (4)
C11—C21	1.376 (4)	O23—C13	1.239 (4)
C11—H11	0.9300	O23—Cu1^i^	2.271 (2)
C21—C31	1.382 (4)	C13—C23	1.526 (5)
C21—H21	0.9300	C23—H23A	0.9600
C31—C41	1.388 (4)	C23—H23B	0.9600
C31—C61	1.517 (4)	C23—H23C	0.9600
C41—C51	1.376 (4)	O14—C14	1.280 (4)
C41—H41	0.9300	O24—C14	1.237 (4)
C51—H51	0.9300	C14—C24	1.501 (5)
N12—C52	1.343 (4)	C24—H24A	0.9600
N12—C12	1.345 (4)	C24—H24B	0.9600
N22—C62	1.324 (4)	C24—H24C	0.9600
N22—H22A	0.8600		
			
O13—Cu1—O14	149.31 (9)	N12—C12—C22	123.7 (3)
O13—Cu1—N12	91.88 (10)	N12—C12—H12	118.2
O14—Cu1—N12	91.34 (9)	C22—C12—H12	118.2
O13—Cu1—N11	89.15 (9)	C12—C22—C32	119.3 (3)
O14—Cu1—N11	88.80 (9)	C12—C22—H22	120.3
N12—Cu1—N11	177.73 (10)	C32—C22—H22	120.3
O13—Cu1—O23^i^	123.64 (10)	C22—C32—C42	117.3 (3)
O14—Cu1—O23^i^	86.76 (9)	C22—C32—C62	124.2 (3)
N12—Cu1—O23^i^	91.51 (9)	C42—C32—C62	118.5 (3)
N11—Cu1—O23^i^	86.23 (9)	C52—C42—C32	119.6 (3)
C51—N11—C11	116.9 (2)	C52—C42—H42	120.2
C51—N11—Cu1	122.7 (2)	C32—C42—H42	120.2
C11—N11—Cu1	119.94 (19)	N12—C52—C42	123.3 (3)
C61—N21—H21A	120.0	N12—C52—H52	118.4
C61—N21—H21B	120.0	C42—C52—H52	118.4
H21A—N21—H21B	120.0	O12—C62—N22	123.6 (3)
N11—C11—C21	123.0 (3)	O12—C62—C32	119.2 (3)
N11—C11—H11	118.5	N22—C62—C32	117.1 (3)
C21—C11—H11	118.5	C13—O13—Cu1	129.2 (2)
C11—C21—C31	119.7 (3)	C13—O23—Cu1^i^	146.6 (2)
C11—C21—H21	120.1	O23—C13—O13	125.9 (3)
C31—C21—H21	120.1	O23—C13—C23	119.3 (3)
C21—C31—C41	117.5 (3)	O13—C13—C23	114.8 (3)
C21—C31—C61	118.8 (3)	C13—C23—H23A	109.4
C41—C31—C61	123.7 (3)	C13—C23—H23B	109.8
C51—C41—C31	119.3 (3)	H23A—C23—H23B	109.5
C51—C41—H41	120.3	C13—C23—H23C	109.2
C31—C41—H41	120.3	H23A—C23—H23C	109.5
N11—C51—C41	123.4 (3)	H23B—C23—H23C	109.5
N11—C51—H51	118.3	C14—O14—Cu1	108.03 (19)
C41—C51—H51	118.3	O24—C14—O14	122.6 (3)
O11—C61—N21	123.9 (3)	O24—C14—C24	120.3 (3)
O11—C61—C31	119.6 (3)	O14—C14—C24	117.1 (3)
N21—C61—C31	116.6 (3)	C14—C24—H24A	109.2
C52—N12—C12	116.7 (2)	C14—C24—H24B	109.6
C52—N12—Cu1	123.6 (2)	H24A—C24—H24B	109.5
C12—N12—Cu1	119.5 (2)	C14—C24—H24C	109.7
C62—N22—H22A	120.0	H24A—C24—H24C	109.5
C62—N22—H22B	120.0	H24B—C24—H24C	109.5
H22A—N22—H22B	120.0		

Symmetry codes: (i) −*x*+1, −*y*+1, −*z*+1.

### Hydrogen-bond geometry (Å, °)

**Table:**

*D*—H···*A*	*D*—H	H···*A*	*D*···*A*	*D*—H···*A*
N21—H21A···O12^ii^	0.86	2.03	2.884 (3)	174
N21—H21B···O14^iii^	0.86	2.15	2.946 (3)	154
N22—H22A···O11^iv^	0.86	2.10	2.955 (3)	174
N22—H22B···O24^v^	0.86	2.19	3.044 (4)	172
C22—H22···O24^v^	0.93	2.26	3.154 (4)	160
C11—H11···O23^i^	0.93	2.56	3.047 (4)	113
C12—H12···O23^i^	0.93	2.53	3.054 (4)	116
C51—H51···O13	0.93	2.36	2.910 (4)	117
C52—H52···O13	0.93	2.57	3.017 (4)	110

Symmetry codes: (ii) *x*+1, *y*, *z*+1; (iii) *x*, −*y*+3/2, *z*+1/2; (iv) *x*−1, *y*, *z*−1; (v) *x*, −*y*+3/2, *z*−1/2; (i) −*x*+1, −*y*+1, −*z*+1.
